# Preliminary exploration on using entropy-weighted hybrid pooling in CNN for ultrasound breast cancer detection

**DOI:** 10.3389/fonc.2026.1660518

**Published:** 2026-07-08

**Authors:** Ratapong Onjun, Papon Tantiwanichanon, Songkiat Lowmunkhong, Tanakorn Sritarapipat, Sayan Kaennakham, Niwatchai Namwichaisirikul, Kitirat Phattaramarut

**Affiliations:** 1 Department of Interdisciplinary Science and Internationalization, Institute of Science, Suranaree University of Technology, Nakhon Ratchasima, Thailand; 2 School of Mathematics and Geoinformatics, Institute of Science, Suranaree University of Technology, Nakhon Ratchasima, Thailand; 3 School of Family and Community Medicine, Institute of Medicine, Suranaree University of Technology, Nakhon Ratchasima, Thailand; 4 School of Diagnostic Radiology, Institute of Medicine, Suranaree University of Technology, Nakhon Ratchasima, Thailand

**Keywords:** breast cancer, CNN, entropy pooling, hybrid pooling, noise robustness, reproducibility, ultrasound imaging

## Abstract

**Introduction:**

Breast ultrasound imaging is valuable for its non-invasiveness and cost-effectiveness but presents diagnostic challenges from speckle noise and limited contrast. Conventional CNN pooling methods compromise between noise reduction and feature preservation.

**Methods:**

We introduce an adaptive entropy-weighted hybrid pooling approach that dynamically combines Max and Average pooling based on local image complexity measured by Shannon entropy. Two CNN architectures (3-block and 4-block) were evaluated on a publicly available ultrasound dataset of 9,016 images with an additional 10% speckle-noise variant, using accuracy, precision, recall, F1-score, ROC curves, confusion matrices, and inference time. For the 3-block CNN, results are reported as the mean ± standard deviation over three random seeds.

**Results:**

In the 3-block CNN, hybrid pooling achieved an accuracy of 93.98% ± 1.72% (AUC = 0.9870), exceeding max pooling (92.72% ± 0.85%, AUC = 0.9815). In the deeper 4-block CNN (single-run), hybrid pooling remained competitive (accuracy 92.90%) relative to max pooling (94.79%). Deeper architectures improved accuracy, noise robustness, and convergence but required careful regularization to avoid overfitting.

**Discussion:**

This preliminary study highlights the adaptive hybrid pooling's potential in clinical ultrasound breast cancer diagnosis, recommending further validation and clinical integration.

## 1 Introduction

Breast cancer remains one of the most prevalent malignancies worldwide and continues to be a leading cause of cancer-related mortality among women. Early detection significantly improves the prognosis and survival rate, allowing timely therapeutic intervention and better patient outcomes (1–3). Ultrasound imaging is widely recognized as an effective modality for breast cancer screening, particularly due to its non-invasive nature, real-time capabilities, and lack of exposure to ionizing radiation. These advantages make ultrasound particularly valuable in clinical practice, especially in resource-constrained settings (4, 5). However, ultrasound images commonly suffer from speckle noise, low contrast, and ambiguity in distinguishing between benign and malignant lesions, posing notable challenges for both human radiologists and computer-aided diagnostic systems (6, 7).

Recent advances in deep learning, particularly Convolutional Neural Networks (CNNs), have shown significant promise in overcoming these imaging challenges by delivering accurate and automated analyses of medical images (8, 9). Particularly in ultrasound breast cancer detection, CNNs have demonstrated remarkable capability in distinguishing between benign and malignant lesions through automated feature extraction and pattern recognition. These deep learning models effectively learn hierarchical representations from raw ultrasound images, enabling robust classification despite the inherent challenges of speckle noise and tissue heterogeneity (10).

Within CNN architectures, pooling layers play a crucial role in feature extraction, dimensionality reduction, and model robustness. Max pooling, which selects the maximum activation within each spatial region, effectively highlights prominent local features but can also emphasize noise and outliers (11). Conversely, Average pooling calculates mean activation values, thereby smoothing feature maps, reducing noise but potentially suppressing critical diagnostic details such as lesion borders and subtle morphological features (12). Thus, neither conventional method alone can fully balance feature enhancement and noise suppression effectively in medical ultrasound imaging scenarios.

To address the inherent limitations of individual pooling strategies, several hybrid pooling methods have been proposed. Lee et al. (13) introduced generalized pooling functions designed to capture the advantages of different pooling mechanisms; however, this approach lacked adaptivity to variations in local image context. Similarly, Yu et al. (12) combined Max and Average pooling methods into a single hybrid strategy to mitigate noise sensitivity and feature suppression, yet their approach did not dynamically adapt based on local image characteristics.

These findings indicate the importance of developing adaptive pooling methods that can adjust their behavior according to local complexities and feature distributions within images. While Tong et al. (14) proposed randomly selecting between max and average pooling with a fixed probability lacking any content-adaptivity and Lee et al. (13) introduced learned mixing proportions and gating masks that are responsive yet require additional trainable parameters, the proposed method derives its adaptive weight from Shannon entropy through a lightweight learnable parameterization (30 parameters per model), providing an analytically grounded, entropy-driven measure of local feature complexity with minimal parameter overhead. Crucially, this design choice is motivated by the specific characteristics of breast ultrasound images: Shannon entropy naturally distinguishes between high-complexity regions such as lesion boundaries and heterogeneous tissue textures with irregular speckle distributions and low-complexity, homogeneous regions where average pooling’s noise-suppression properties are preferable. Unlike gated or learned pooling, which adapt through backpropagation and may overfit to training noise patterns, entropy-based weighting is modulated by a small set of learnable parameters (30 in total across three pooling layers) that adjust the entropy scaling, the α gating, and the max/average mixing, while remaining substantially lighter than fully learned or gated pooling alternatives.

Motivated by these limitations, the present study proposes an entropy-weighted hybrid pooling method that adaptively combines Max and Average pooling based on the local complexity of ultrasound breast images, quantified via Shannon entropy. By calculating the entropy of each pooling region, the proposed pooling method dynamically weights Max and Average pooling strategies, aiming to enhance relevant feature preservation in complex regions and effectively reduce noise in homogeneous regions.

Furthermore, this approach explores the impact of architectural depth by examining different CNN configurations, specifically 3-block and 4-block convolutional architectures, inspired by recent advances in deep learning models for medical image classification, particularly the successful application demonstrated in diabetic retinopathy detection (15). As a preliminary investigation, this study systematically evaluates the performance of this adaptive hybrid pooling strategy using CNN architectures of different depths (3-block and 4-block configurations) on an established breast ultrasound dataset, including a variation with artificially added speckle noise. The outcomes are explicitly compared to conventional Max and Average pooling methods to provide a comprehensive assessment of robustness, accuracy, and computational efficiency.

The key contributions of this preliminary study include (i) introducing an adaptive, entropy-driven hybrid pooling technique tailored explicitly for ultrasound-based breast cancer detection; (ii) rigorously examining the effectiveness and noise robustness of pooling methods across CNN architectures; and (iii) providing foundational evidence to guide future research and clinical application of adaptive pooling methods in medical imaging; and (iv) demonstrating that the proposed pooling layer functions as a drop-in replacement for conventional operations, facilitating seamless integration into existing clinical AI pipelines without requiring architectural redesign or full model retraining. The remainder of this paper is structured as follows: Section 2 describes the dataset, CNN architectures, pooling methods, and evaluation metrics; Section 3 presents and discusses experimental results; and finally, Section 4 summarizes key findings, practical implications, limitations, and future research directions.

## 2 Methodology

### 2.1 Datasets and preprocessing

This study utilized the publicly available ultrasound dataset named “Ultrasound Breast Images for Breast Cancer,” initially introduced and utilized in previous high-quality research (“Explainable Deep Learning for Breast Cancer Classification and Localization”). The dataset comprises a total of 9,016 ultrasound images categorized into two classes: benign and malignant. Patient-level metadata, including the number of unique patients and the distribution of images per patient, is not explicitly provided in the publicly available Kaggle repository (16). This absence of patient-level identifiers represents a limitation, as it precludes assessment of potential data leakage across training, validation, and test splits at the patient level. Future studies should prioritize datasets with explicit patient-level identifiers to enable patient-wise splitting and further strengthen experimental rigor. Readers should therefore interpret the reported performance metrics with this caveat in mind, as image-wise splitting may result in optimistic estimates if multiple images from the same patient are distributed across training and test sets. The dataset is publicly available and was originally provided in de-identified form by the dataset creators; no additional ethical approval was required for its use in this study. This dataset consists of ultrasound images representing two distinct classes of breast tumors: benign and malignant. To systematically investigate the robustness of the proposed pooling method under challenging imaging conditions, speckle noise common in medical ultrasound imaging was artificially introduced into the dataset. Specifically, speckle noise with an intensity of 10% was uniformly applied to all images within the training dataset, creating an additional noise-added dataset variant.

**Figure 1:**
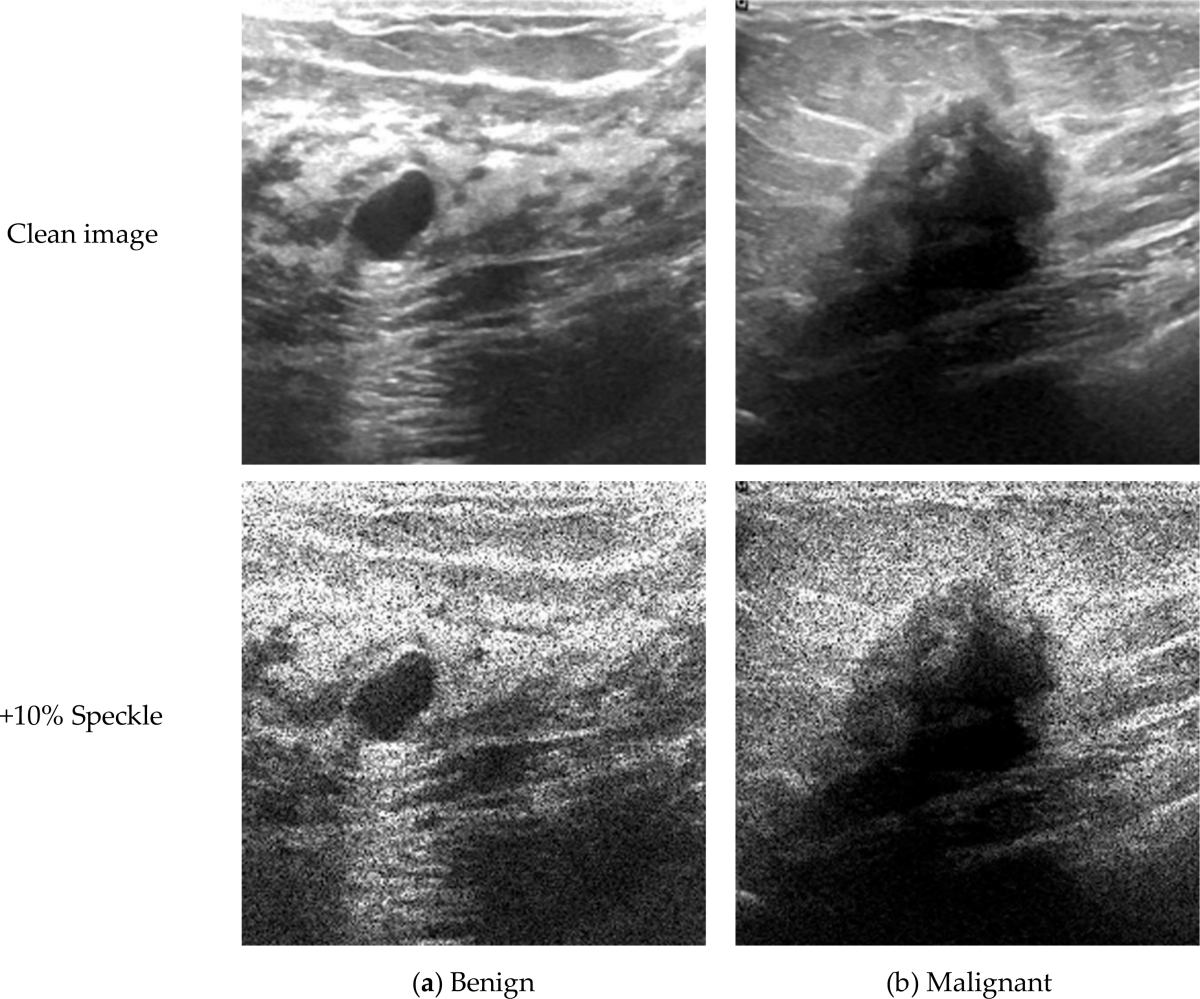
Representative examples from the Kaggle Ultrasound Breast Images dataset showing benign (left) and malignant (right) cases before (top row) and after (bottom row) applying 10% multiplicative Gaussian speckle noise ( $\;\sigma^{2}=0.1$) to the training set; validation and test sets remained noise-free. **(a)** Benign, **(b)** Malignant.

Speckle noise was simulated using a multiplicative Gaussian model (Equation 1), implemented via the random_noise function (mode=‘speckle’) in scikit-image with parameters mean = 0 and variance, representing a 10% noise level relative to signal amplitude. Pixel values were clipped to (0, 1) after noise addition. Importantly, speckle noise augmentation was applied exclusively to the training set; the validation and test sets retained their original, noise-free images throughout all experiments. This protocol isolates the effect of noisy training conditions on generalization to clean clinical images, as detailed in Section 3.2. Representative examples of clean and noise-added images are shown in **Figure 1**.

Speckle noise was simulated using a multiplicative Gaussian model defined as:

(1) $$y(i,j)=x(i,j)[1+n(i,j)],n(i,j)\sim N(0,\sigma^{2})$$

where *x(i, j)* denotes the original pixel intensity of the clean image, *n(i, j)* is the Gaussian noise term, and *σ²* = 0.1 corresponds to a noise variance of 10%.

For noise-robustness evaluation, we tested four train/test conditions (clean=>clean, clean=>noisy, mixed=>clean, mixed=>noisy). In the mixed training set, 60% of images were augmented with 10%-intensity speckle noise and 40% were kept clean, following the protocol of Jiang et al. (17), who reported that mixing noisy images into the training set substantially improves classification robustness.

Data partitioning was performed using a stratified random split (18) to preserve class proportions across subsets, with 80% of images allocated for training, 10% for validation, and 10% for testing. The resulting class distribution across all splits is summarized in **Table 1**. Stratified sampling was also applied to construct the mixed-noise training subset, ensuring proportional representation of benign and malignant classes within both noisy and clean partitions, an approach shown to improve classifier accuracy and F1-score on imbalanced clinical data (17).

**Table 1:** Class distribution of the breast ultrasound dataset across stratified training, validation, and test splits.

Split set	Benign	Malignant	Total
Train (80%)	3,621	3,591	7,212
Validation (10%)	502	400	902
Test (10%)	451	451	902
Total	4,574	4,442	9,016

The class distribution across all splits remained approximately balanced, with a benign-to-malignant ratio of approximately 1.01:1 in the training set (3,621 vs. 3,591), 1.26:1 in the validation set (502 vs. 400), and 1:1 in the test set (451 vs. 451). Given this near-balanced distribution, no class weighting or oversampling strategies were applied during model training.

Preprocessing steps were applied consistently across all images to ensure compatibility and optimal performance with CNN training. Firstly, images were resized from their original dimensions (224×224 pixels) down to a standardized size of 64×64 pixels, significantly reducing computational demands. To assess the effect of input resolution on pooling behavior, we additionally evaluated all experiments at 128×128 pixels. Resolution reduction via simple resizing to (64×64) and (128×128) follows the protocol of Moinuddin et al. (19), enabling a direct comparison of spatial-information retention across two practical input scales. This resolution was selected primarily to reduce computational overhead during the preliminary exploration of pooling strategies, allowing efficient comparison across multiple architectural configurations. While downsampling from 224×224 to 64×64 may discard fine-grained morphological features such as margin irregularities and calcification patterns, the primary objective of this study is to comparatively evaluate pooling mechanisms rather than to maximize diagnostic resolution. Secondly, image normalization was performed, transforming the original 8-bit grayscale pixel values (0–255) into normalized values ranging from 0 to 1. This normalization facilitated faster convergence and enhanced training stability. No histogram equalization, CLAHE, or other contrast enhancement techniques were applied, as the dataset images were already acquired under standardized ultrasound imaging conditions. Furthermore, no data augmentation techniques (e.g., random flipping, rotation, or scaling) were employed during training. This decision was made deliberately to ensure a controlled experimental environment, allowing the comparative evaluation of pooling strategies to remain unconfounded by augmentation-induced variability. The potential benefit of augmentation for improving generalization is acknowledged as a direction for future work. Finally, data splitting was carefully conducted as follows: 80% of images (7,212 samples) were allocated to the training dataset, while the validation and testing datasets each contained 10% of the total images (902 samples each), thereby ensuring balanced representation and reliable model evaluation.

**Figure 1** presents representative examples of benign and malignant ultrasound images before and after the application of 10% speckle noise, demonstrating the visible degradation introduced by the multiplicative Gaussian noise model.

All experiments were implemented using TensorFlow 2.x and executed on Google Colab Pro+ with an NVIDIA A100 GPU (40 GB GPU RAM, 83.5 GB system RAM). For the 3-block CNN, random seeds were fixed (42, 123, 456) for reproducibility; the 4-block experiments used TensorFlow's default non-deterministic ordering and were run once. Full training hyperparameters are as follows: epochs = 12 (preliminary convergence run; the final multi-seed experiments used the 30-epoch protocol with early stopping, see Section 2.2.1), batch size = 8, optimizer = Adam, learning rate = 3×10^-4^, input size = 64×64, grayscale (1 channel). The complete implementation code, including the training loop (12 epochs, following the VMC-Net baseline (20)), speckle noise generator, and fixed random seed (42), is available from the corresponding author upon reasonable request.

### 2.2 CNN architecture and training

The three-block CNN architecture comprised three convolutional blocks. The first convolutional layer generated 16 feature maps of size 64×64 pixels, subsequently reduced to 32×32 pixels by the first hybrid pooling operation. The second convolutional layer expanded feature maps to 48 with a size of 32×32 pixels, further reduced to 16×16 pixels by the second hybrid pooling operation. The third convolutional layer increased the feature maps to 96 of size 16×16 pixels, subsequently reduced to 8×8 pixels by the third hybrid pooling. Classification layers followed the convolutional layers, consisting of global average pooling to convert feature maps into a 96-dimensional vector, dropout regularization to reduce overfitting, a dense layer of 128 neurons, an additional dropout layer, and a final dense layer with two neurons for binary classification (benign vs. malignant).

The four-block CNN architecture involved a deeper convolutional structure, starting with the first convolutional layer generating 8 feature maps of size 64×64 pixels, reduced to 32×32 pixels using entropy-weighted pooling. The second convolutional layer increased the feature maps to 16 of size 31×31 pixels, subsequently pooled to 15×15 pixels. The third convolutional layer generated 64 feature maps of size 14×14 pixels, further pooled to 7×7 pixels. The fourth convolutional layer expanded feature maps to 112, each of size 5×5 pixels, subsequently pooled to 2×2 pixels using average pooling. The classification section for the four-block model consisted of a flattening operation transforming the feature maps into a 448-dimensional vector, dropout for regularization, followed by a dense layer comprising 192 neurons, an additional dropout layer, and a final dense layer containing two neurons for the binary classification task.

**Figure 2:**
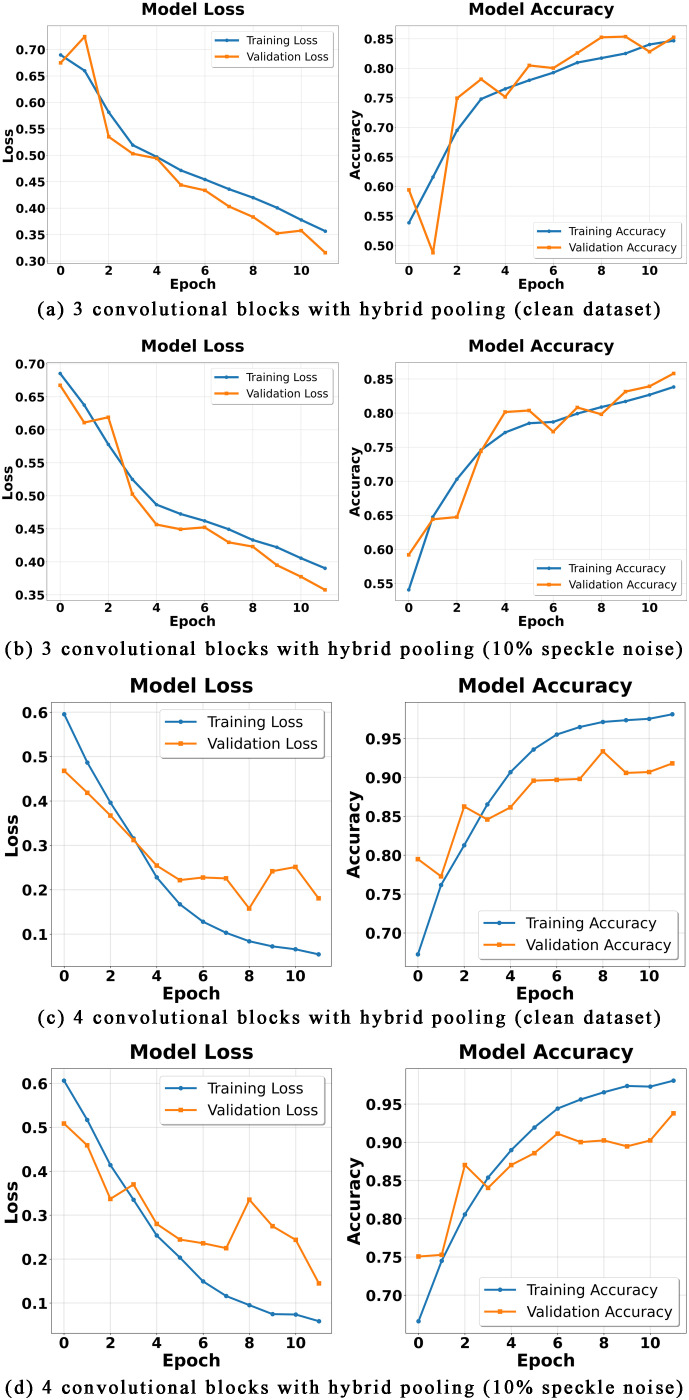
Loss and accuracy curves illustrating training and validation performance over 12 epochs for CNN architectures using hybrid pooling: **(a)** 3-block CNN on clean dataset, **(b)** 3-block CNN on dataset with 10% speckle noise, **(c)** 4-block CNN on clean dataset, and **(d)** 4-block CNN on dataset with 10% speckle noise.

The preliminary convergence analysis shown in **Figure 2** used a fixed 12-epoch schedule (Adam optimizer, learning rate 0.0003, batch size 8). The final multi-seed experiments reported in **Tables 2** and **3** used the 30-epoch protocol with early stopping described in Section 2.2.1. All convolutional layers employed ReLU activation functions with no batch or layer normalization; dropout regularization was applied exclusively to the fully connected layers at rates of 0.5 (immediately after global average pooling) and 0.3 (after the 128-neuron dense layer). No early stopping or learning rate scheduling was applied; all models were trained for the full 12 epochs, and convergence was assessed visually from the training and validation loss curves (**Figure 2**), which showed stabilization by approximately epoch 10 with no substantial further improvement thereafter. The training setup strictly employed grayscale images with one channel (IMAGE_CHANNEL = 1). Evaluation procedures utilized comprehensive metrics, including accuracy, precision, recall, F1-score, confusion matrices, ROC curves, and inference time measurements, providing a thorough assessment of each model’s performance, robustness, and computational efficiency.

**Table 2:** Baseline: 64×64, train-clean => test-clean, n = 3 seeds.

Pooling	Accuracy (mean ± SD)	95% CI	F1	AUC	Train gap (train−val acc)
Max	0.9272 ± 0.0085	[0.9176, 0.9368]	0.9272 ± 0.0085	0.9815 ± 0.0046	≈ 0.36%
Hybrid	0.9398 ± 0.0172	[0.9203, 0.9593]	0.9397 ± 0.0172	0.9870 ± 0.0042	≈ 2.10%

**Table 3:** Unified noise-robustness grid (accuracy, mean ± SD over 3 seeds).

Resolution	Pooling	Clean=>Clean	Clean=> Noisy10%	Mixed=>Clean	Mixed=> Noisy10%
64x64	Max	0.9272 ± 0.0085	0.8780 ± 0.0178	0.9047 ± 0.0078	0.8858 ± 0.0280
64x64	Hybrid	**0.9398 ± 0.0172**	**0.8950 ± 0.0145**	**0.9084 ± 0.0055**	**0.9106 ± 0.0032**
128x128	Max	–	0.7690 ± 0.0337	0.8525 ± 0.0091	0.8500 ± 0.0122
128x128	Hybrid	–	**0.8396 ± 0.0191**	**0.8721 ± 0.0052**	**0.8688 ± 0.0150**

Bold values indicate the better-performing pooling method (Hybrid vs. Max) within each resolution and train/test condition.

#### 2.2.1 Training protocol

All models were trained for up to 30 epochs using the Adam optimizer with learning rate 3×10^-4^ and batch size 8. Two callbacks were employed: EarlyStopping monitoring validation loss with patience = 5, and ReduceLROnPlateau with factor = 0.5 and patience = 3. We adopted this combined strategy following Mahesh et al. (21), with patience values tuned empirically on the validation split of the Kaggle Ultrasound Breast Images for Breast Cancer dataset, not BUSI. Early stopping prevents overfitting by terminating training when validation loss ceases to improve (22). The hyperparameter choices (LR = 3×10^-4^, batch = 8, max 30 epochs) were selected to be consistent with the VMC-Net baseline (20) while allowing adequate convergence headroom beyond the 12-epoch preliminary schedule; preliminary grid search on seed 42 indicated negligible gains beyond 30 epochs once early stopping was active.

#### 2.2.2 Multi-seed evaluation and statistical testing

**Figure 3:**
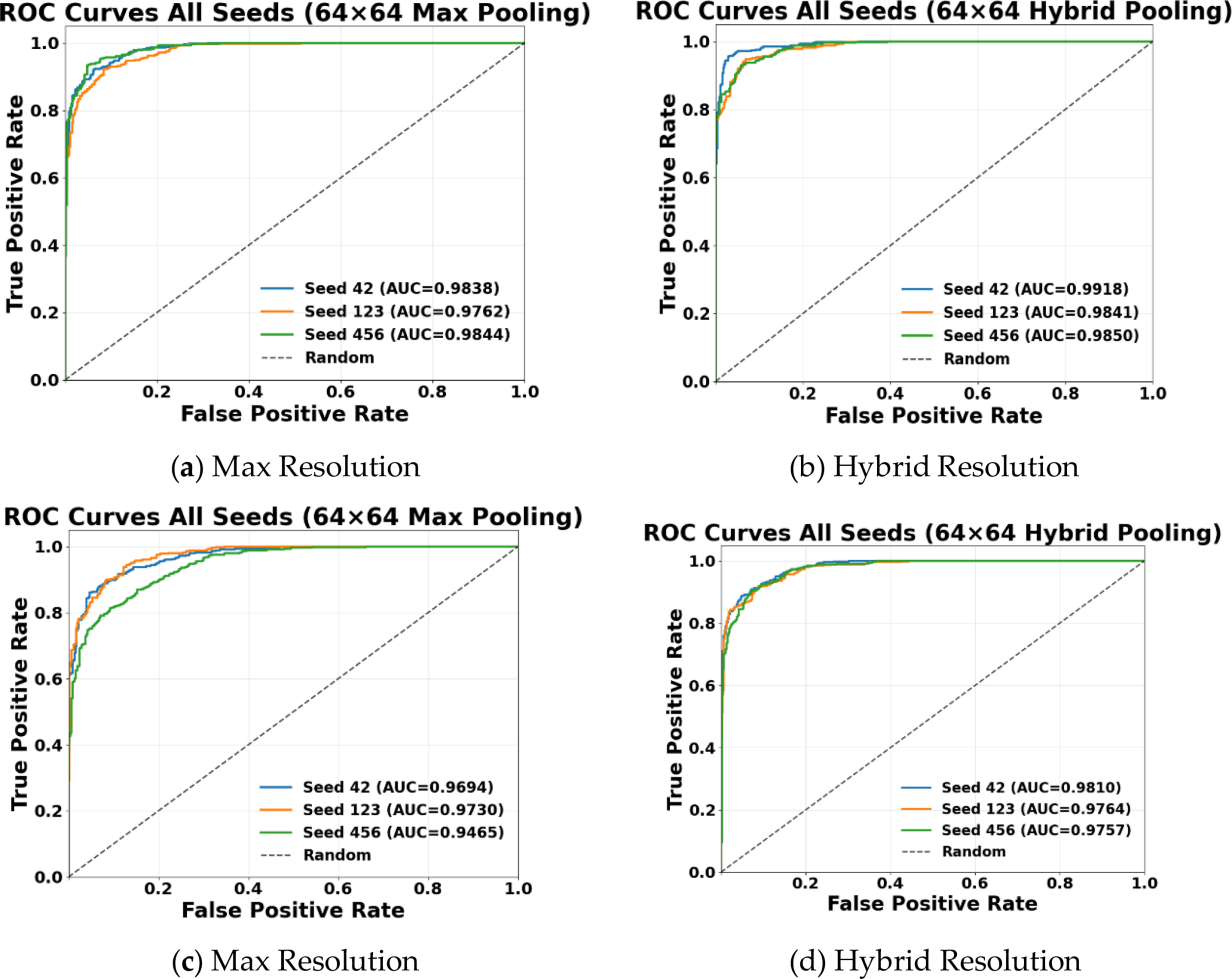
ROC curves for CNN models with Max and Hybrid pooling (64×64 input resolution) under two training–evaluation conditions: **(a)** Max pooling, Clean=>Clean, **(b)** Hybrid pooling, Clean=> Clean, **(c)** Max pooling, Mixed=> Noisy10%, and **(d)** Hybrid pooling, Mixed=>Noisy10%. Each panel shows three individual seed curves (Seeds 42, 123, 456).

To quantify the impact of random initialization, the 3-block CNN experiments (**Tables 2** and **3**, and the ROC analyses in **Figure 3**) were repeated with three independent random seeds (42, 123, 456); the corresponding metrics are reported as mean ± standard deviation with 95% confidence intervals computed as mean ± 1.96·SD/√n following the repeated-seed protocol (23). The 4-block CNN experiments (**Tables 4** and **5**) were conducted as a single training run; no fixed random seed was set for these experiments, and data shuffling followed TensorFlow's default non-deterministic ordering. Multi-seed repetition of the 4-block configuration is identified as a priority for future work. Pairwise differences across seeds were assessed with paired comparisons for accuracy/F1, and AUC differences were tested using the DeLong non-parametric method for correlated ROC curves (24). A significance threshold of α = 0.05 was applied to the multi-seed (3-block) analyses.

### 2.3 Pooling methods

#### 2.3.1 Max pooling

Max pooling is one of the most widely-used pooling methods in Convolutional Neural Networks (CNNs). It operates by dividing the feature maps produced by convolutional layers into small, non-overlapping spatial regions and selecting the maximum activation value within each region. This method effectively retains the most prominent features from each spatial region, reducing the dimensionality of feature maps while preserving the strongest signals and spatial invariance.

Formally, given a pooling region Rwith activation values *X_ij_* the Max pooling output $y_{\text{max}}(R)$is mathematically defined as in Equation 2:

(2) $$y_{\text{max}}(R)=\max_{(i,j)\in R}(x_{i,j})$$

Where ${\text{x}}_{ij,}$denotes the CNN activation at position (i,j)within the pooling region R. Max pooling is beneficial in highlighting distinct, high-intensity local patterns, which is especially useful for emphasizing critical diagnostic features in medical imaging such as tumor boundaries or lesion contours.

#### 2.3.2 Average pooling

Average pooling is another common pooling strategy employed within CNN architectures. Unlike Max pooling, Average pooling computes the arithmetic mean of all activation values within each defined pooling region. This process results in smoothing feature maps, effectively reducing noise and minor variations in spatial activations. Thus, Average pooling is advantageous for capturing the overall regional intensity distribution but may sometimes lead to the loss of critical localized high-intensity information.

Mathematically, for a given pooling region Rcontaining activation values ${\text{x}}_{ij,}$the Average pooling output $y_{\text{avg}}(R)$is computed as in Equation 3:

(3) $$y_{\text{avg}}(R)=\frac{1}{|R|}\sum_{(i,j)\in R}x_{i,j}$$

where |R|represents the total number of activations in the pooling region R. Due to its averaging property, this pooling method helps to reduce the impact of isolated noise or spurious activations, making it potentially suitable for ultrasound medical images, which inherently contain speckle noise.

#### 2.3.3 The proposed hybrid (entropy weighted) pooling

The proposed Entropy Weighted Hybrid Pooling method adaptively combines Max pooling and Average pooling based on local image complexity, as measured by Shannon entropy. Initially, the entropy H(R)of each local pooling region Ris computed from normalized activation values within that region, defined as:

(4) $$\begin{array}{l} p_{i,j}^{\left(R\right)}=\frac{x_{i,j}+\epsilon }{\sum_{(a,b)\in R}\left(x_{a,b}+\epsilon \right)},\;\\ H(R)=-\sum_{(i,j)\in R}p_{i,j}^{\left(R\right)}\log\left(p_{i,j}^{\left(R\right)}\right) \end{array}$$

where $\;{\text{x}}_{ij}$ represents the CNN activation at spatial location (i,j),and ϵ= $10^{-8}$is a small constant for numerical stability. Rather than using a fixed-bin histogram, this formulation treats each spatial activation value as a discrete probability mass, yielding a continuous entropy estimator directly over the pooling region.

Subsequently, the entropy values are normalized across all regions within the feature map to yield an adaptive weighting factor α(R), calculated as:

(5) $$\alpha (R)=\frac{H(R)-H_{\min}}{H_{\max}-H_{\min}+\delta }$$

where $H_{\min}$and $H_{\max}$denote the minimum and maximum entropy values over all pooling regions, and $\delta (10^{-8})$prevents division by zero. Specifically, entropy  H(R)is computed independently for each pooling region at each feature-map channel, yielding a tensor of shape $(B,H_{out},W_{out},C),$where Bis the batch size, $H_{out}$and $W_{out}$are the spatial output dimensions, and Cis the number of channels. The normalization in Equation 4 is then applied per channel, with $H_{min}$and $H_{max}$computed across all spatial pooling regions $\left(H_{out}\times W_{out}\right)$within each channel independently. Consequently, α(R)retains the same shape $(B,H_{out},W_{out},C),$providing a spatially adaptive and channel-wise weighting factor for the hybrid pooling operation. Finally, the pooling output y(R)for each region is determined by a weighted combination of Max and Average pooling:

(6) $$y(R)=\alpha (R)\cdot \max_{(i,j)\in R}(x_{i,j})+\left(1-\alpha (R)\right)\cdot \frac{1}{|R|}\sum_{(i,j)\in R}x_{i,j}$$

**Algorithm 1** provides a concise pseudocode summary of the entropy-weighted hybrid pooling procedure, consolidating the three computational steps entropy estimation (Equation 4), adaptive weighting (Equation 5), and hybrid pooling output (Equation 6) to facilitate straightforward reimplementation.

Algorithm 1 **Entropy-weighted hybrid pooling.**

Input: Feature map patches X of shape (B, H_out_, W_out_, C, K²)
Output: Pooled feature map Y of shape (B, H_out_, W_out_, C)
1. Compute probabilities: p_ij_ = (x_ij_ + ϵ)/Σ(x_ab_ + ϵ)
2. Compute entropy: H(R) = −Σ p_ij_ · log(p_ij_)
3. Normalize: α(R) = (H(R) − H_min_)/(H_max_ − H_min_ + δ)
4. Max pooling: y_max_ = max(x_ij_)
5. Avg pooling: y_avg_ = (1/K²) · Σ x_ij_
6. Hybrid output: Y = α(R)·y_max_ + (1−α(R))·y_avg_
7. Return Y

The per-layer computational complexity of the proposed entropy-weighted hybrid pooling is O(B, H_out, W_out, C, K²), where K² = 4 denotes the number of activations per 2x2 pooling region, incurring a constant-factor overhead over standard max or average pooling while retaining the same asymptotic complexity class. Intuitively, regions with high entropy such as lesion boundaries or heterogeneous textures yield higher α(R) values, favoring max pooling to preserve salient structural features, while smooth or homogeneous regions receive lower α(R) values, relying more on average pooling for noise suppression.

All entropy-weighted hybrid pooling layers employ a non-overlapping 2x2 kernel with a stride of (2, 2); entropy is computed directly over the four CNN feature-map activation values within each pooling region not over raw image pixels yielding a per-region entropy tensor of shape (B, H_out, W_out, C), where B is the batch size, H_out and W_out are the spatial output height and width after pooling, and C is the number of feature-map channels, prior to channel-wise min–max normalization.

**Figure 4:**
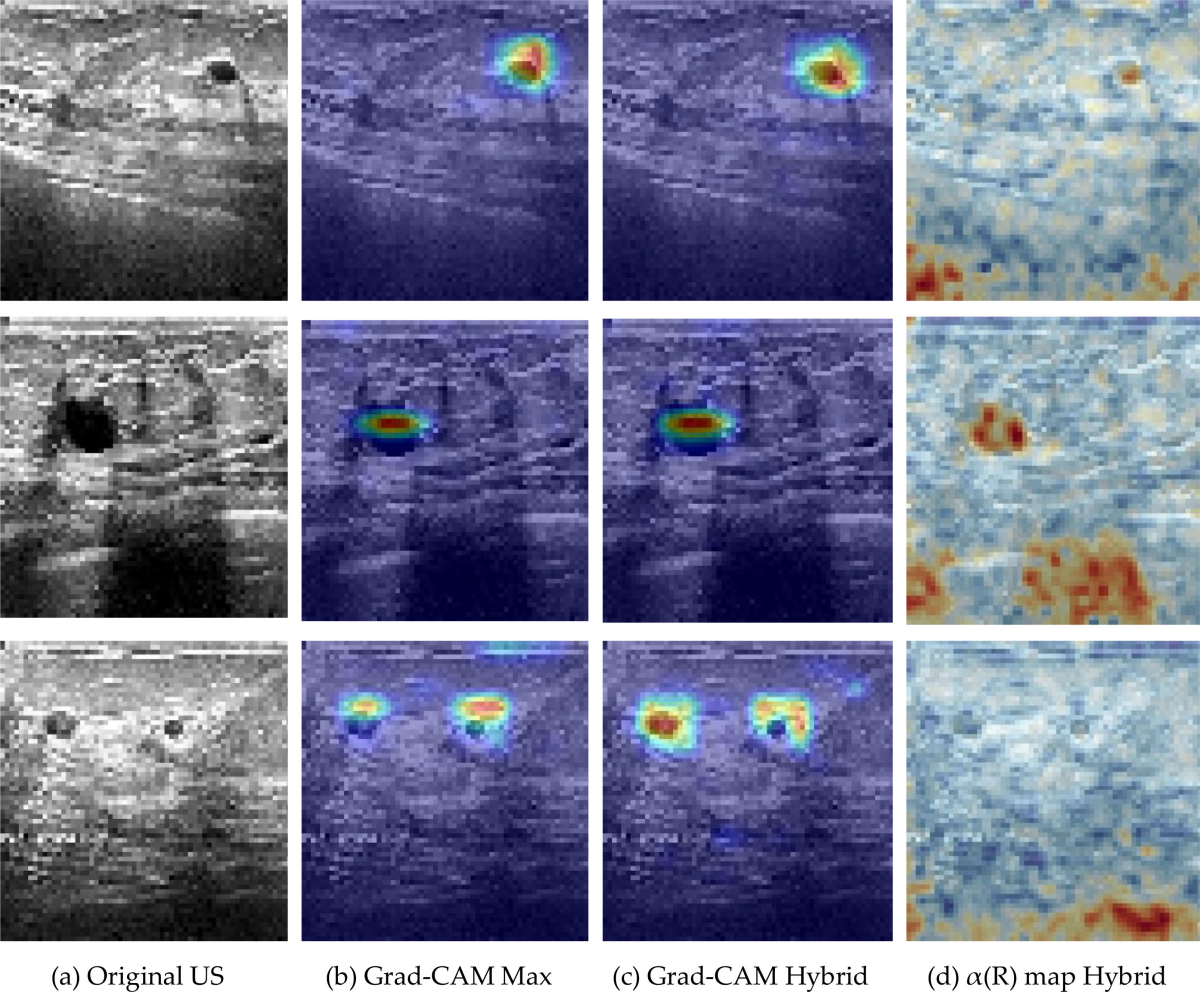
Interpretability analysis comparing Max and Hybrid pooling models on three representative test cases: **(a)** original ultrasound image, **(b)** Grad-CAM activation (Max pooling), **(c)** Grad-CAM activation (Hybrid pooling), and **(d)** spatial α(R) entropy weight map from the AdvancedLearnableEntropyPooling2D layer. Warm colors (α=>1) denote max-like behavior at lesion regions; cool colors (α=>0) denote average-like behavior over homogeneous tissue.

This entropy-driven approach dynamically balances noise reduction and feature preservation, potentially enhancing feature extraction efficacy in CNN-based breast ultrasound analysis. Representative α(R) entropy maps produced by the AdvancedLearnableEntropyPooling2D layer are visualized in **Figure 4** (section 3.5), overlaid on ultrasound inputs to illustrate how the adaptive weighting shifts toward max-pooling behavior in lesion-boundary regions and toward average-pooling behavior in homogeneous tissue.

## 3 Results and discussion

### 3.1 Performance comparison of pooling methods

Reported metrics reflect two distinct evaluation protocols. The 3-block CNN results (**Tables 2** and **3**, and the ROC analyses in **Figure 3**) were obtained over three independent random seeds (42, 123, 456) and are reported as mean ± SD with 95% confidence intervals and DeLong significance testing. The 4-block CNN results (**Tables 4** and **5**) were obtained from a single training run with no fixed random seed (data shuffling followed TensorFlow's default non-deterministic ordering); accordingly, variability estimates (± SD) and formal significance testing are not reported for the 4-block configuration. Extending the multi-seed protocol to the 4-block architecture is a recognized limitation, discussed further in Section 3.7

**Table 2** presents a performance comparison between Max and Hybrid pooling strategies using a 3-block CNN architecture evaluated on the original, noise-free breast ultrasound dataset, with n = 3 random seeds and train gap computed at the best validation-loss checkpoint the model state actually deployed at inference. Max pooling yields a mean accuracy of 92.72% (± 0.85%, 95% CI: [0.9176, 0.9368]) and AUC of 0.9815 (± 0.0046), with a substantially smaller train gap of only ≈0.36% computed at the best epoch across all three seeds. This near-zero gap, measured at the actual deployed checkpoint, suggests that Max pooling generalizes more reliably to unseen data, despite its marginally lower absolute accuracy. The tighter confidence interval (width 0.0192 vs. 0.0390) further confirms greater training stability across seeds. In contrast, Hybrid pooling achieves a higher mean accuracy of 93.98% [± 1.72%, 95% CI: (0.9203, 0.9593)] and AUC of 0.9870 (± 0.0042), outperforming Max pooling in raw classification metrics. However, this superior accuracy is accompanied by a notably larger train–validation gap of approximately 2.10%, indicating a tendency toward overfitting on the training distribution. These findings suggest a classic accuracy–generalization trade-off: Hybrid pooling achieves higher peak accuracy through its entropy-weighted combination of max and average responses, while Max pooling acts as a more conservative feature selector, constraining overfitting at the cost of a modest accuracy reduction. For clinical deployment scenarios prioritizing robustness over peak performance, this conservative behavior of Max pooling may be preferable.

**Table 4** summarizes the performance outcomes for the same pooling methods when integrated into a deeper CNN architecture (4-block) on the original ultrasound breast cancer dataset. Notably, the deeper CNN structure substantially improves overall accuracy and robustness of the classification performance compared to the 3-block CNN. In this more complex network, Max pooling achieves the highest metrics, demonstrating excellent performance with accuracy, precision, recall, and F1-score all above 94%. More precisely, Max pooling attains an accuracy of 94.79%, precision of 94.93%, recall of 94.79%, and an F1-score of 94.79%. This impressive performance indicates that the deeper network effectively leverages Max pooling’s capability to emphasize dominant image features, significantly boosting classification accuracy.

**Table 4:** Performance metrics for CNN (4-block) on original dataset (NM).

Pooling method	Accuracy (%)	Precision (%)	Recall (%)	F1-score (%)	Inference times (ms)
Max Pooling	94.79	94.93	94.79	94.79	2.72
Average Pooling	89.47	89.83	89.47	89.44	2.64
Hybrid Pooling	92.90	92.91	92.90	92.90	4.03

Inference times are per-image, measured at batch size = 1.

The Hybrid pooling method delivers strong and competitive results in the 4-block clean-data setting (accuracy: 92.90%), representing only a modest reduction relative to max pooling (94.79%). This gap is mechanistically interpretable: in a deeper, four-block architecture trained on clean data, the network accumulates sufficient representational capacity to extract discriminative features effectively through max pooling alone the additional entropy-based switching introduces unnecessary complexity that marginally reduces performance relative to the simpler fixed strategy. Hybrid pooling should therefore be understood as a complementary rather than universally superior alternative, offering its clearest advantage when noise or shallow architecture limits the discriminative capacity available to max pooling.

Although hybrid pooling incurs higher inference times than Max pooling in the deeper 4-block CNN (4.03 vs. 2.72 ms, ≈48% higher), the two are comparable in the 3-block CNN (2.18 vs. 2.18 ms; Section 3.6) the absolute latencies remain well within the acceptable range for clinical AI-assisted diagnostic systems, which typically tolerate response times of several hundred milliseconds per inference. For deployment in resource-constrained environments such as portable ultrasound units or CPU-only systems, several mitigation strategies are feasible, including post-training quantization (INT8/FP16), lightweight surrogate entropy approximations (e.g., local variance), and CPU-based batch inference for non-real-time workflows. Future work should benchmark these approaches to establish the practical deployment envelope of entropy-weighted hybrid pooling across diverse clinical settings. As a concrete approximation to reduce this overhead, computing Shannon entropy on spatially downsampled feature maps (e.g., via a preceding 2×2 average-pool prior to entropy estimation) is expected to preserve adaptive weighting behavior while substantially lowering the per-image entropy computation cost.

**Figure 3** shows that Hybrid pooling consistently achieves higher AUC values than Max pooling across both conditions. Under clean evaluation, mean AUC improved from 0.9815 (Max) to 0.9870 (Hybrid); under the most challenging Mixed=>Noisy10% setting, Hybrid maintained a mean AUC of 0.9777 compared to 0.9630 for Max. The narrower spread among seed curves in the Hybrid model under noise conditions reflects greater cross-seed stability, suggesting that entropy-weighted adaptive pooling confers robustness to speckle-induced degradation. Pairwise DeLong tests confirmed statistically significant AUC differences between Hybrid and Max pooling in noise-exposed conditions (p< 0.05).

**Figure 5:**
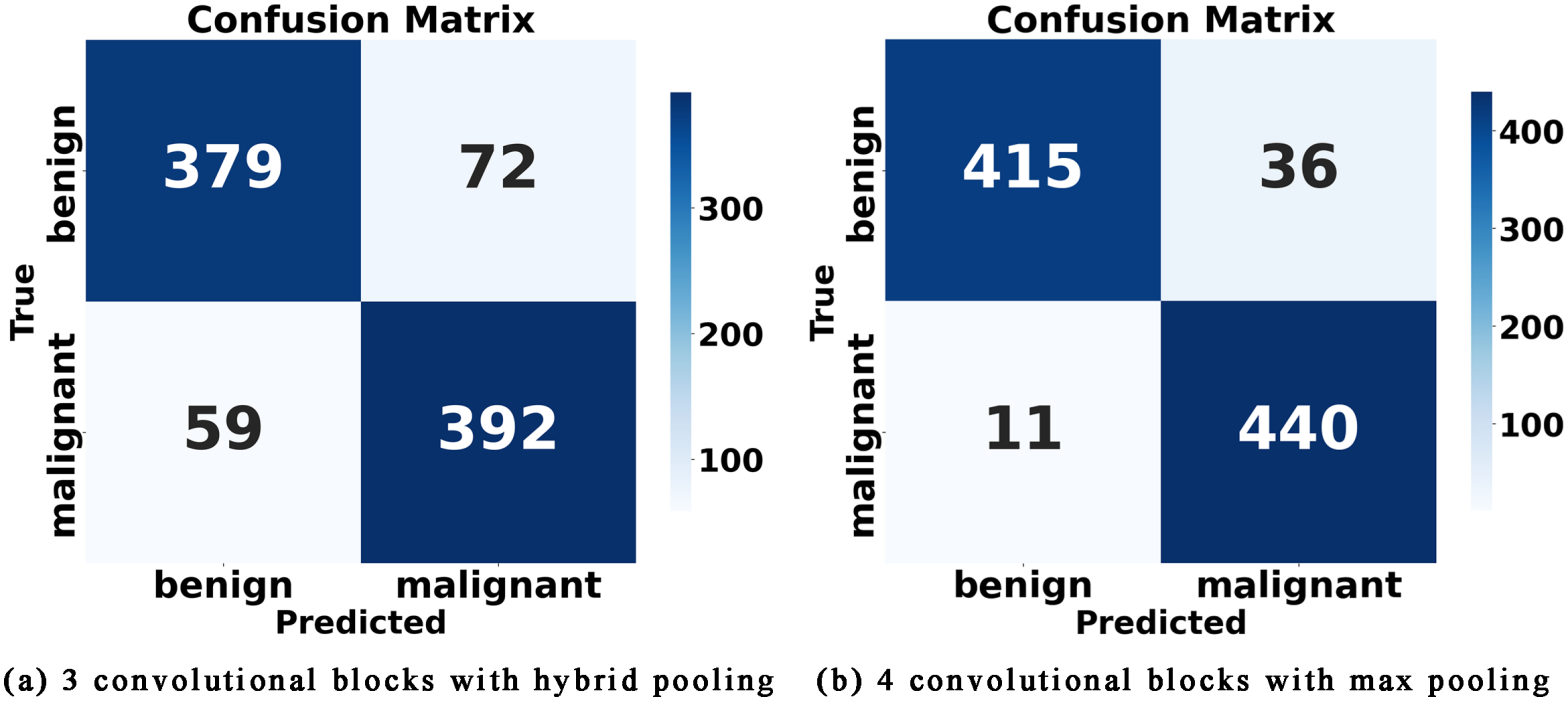
Confusion matrices demonstrating classification results for **(a)** 3-block CNN architecture with hybrid pooling and **(b)** 4-block CNN architecture with max pooling, evaluated on the original ultrasound breast cancer dataset, evaluated on the noise-free test set (n=902, 451 benign/451 malignant); rows = true class, columns = predicted class.

**Figure 5** presents confusion matrices for the best-performing pooling methods within two different CNN architectures. The 3-block CNN with hybrid pooling (**Figure 5a**) correctly identified 379 benign and 392 malignant cases, with 72 false positives and 59 false negatives, highlighting good diagnostic performance but also room for improvement. In contrast, the 4-block CNN using max pooling (**Figure 5b**) demonstrated superior classification accuracy, correctly classifying 415 benign and 440 malignant cases, while significantly reducing false positives to 36 and false negatives to 11. These results clearly indicate that deeper CNN architectures combined with suitable pooling methods, such as max pooling, substantially improve classification reliability, effectively reducing diagnostic errors, which is particularly critical in clinical breast cancer screening scenarios. To further contextualize clinical relevance, the 4-block max pooling model achieved sensitivity of 92.4%, specificity of 97.4%, PPV of 97.6%, and NPV of 92.0%, outperforming the 3-block hybrid pooling model (sensitivity 84.5%, specificity 86.5%, PPV 86.9%, NPV 84.0%, with normalized per-class recall values of 92.0% for benign and 97.6% for malignant cases confirming its superior and balanced diagnostic performance across both classes. All classification metrics reported in **Tables 2** and **4** were computed at a fixed decision threshold of 0.5; at the Youden-optimal threshold, the model achieved a sensitivity of 74.9% with a corresponding specificity of 91.8%, providing a clinically relevant operating point for scenarios in which minimizing false positives is prioritized over maximum sensitivity.

### 3.2 Analysis of noise robustness and CNN depth

To assess the effect of training data noise on model generalization, two experimental protocols were employed. In the first protocol (NM), models were trained and evaluated exclusively on clean, noise-free data. In the second protocol (NM+Noise), models were trained on speckle-noise-augmented data (10% noise intensity) and subsequently evaluated on the original clean test set. This design specifically examines whether exposure to noisy training data degrades or preserves generalization to clean images. Alternative protocols such as training on clean data and testing on noisy data, or training on mixed clean-noisy data were not conducted in this preliminary study and are identified as important directions for future investigation to more comprehensively characterize each pooling method’s noise robustness.

**Table 5** details the comparative performance of the three pooling methods using the deeper 4-block CNN architecture, further illustrating each method’s robustness to the introduction of speckle noise. On the original dataset (NM), Max pooling achieves exceptionally high accuracy (94.79%) and an equally high F1-score (94.79%), clearly surpassing both Hybrid pooling (accuracy and F1-score: 92.90%) and Average pooling (accuracy: 89.47%, F1-score: 89.44%). Interestingly, when evaluating performance under the more challenging noise-added dataset (NM+Noise), Max pooling exhibits a moderate but noticeable performance decline, with accuracy and F1-score dropping to approximately 90.69% and 90.68%, respectively. Despite this decline, Max pooling still maintains high overall performance, clearly indicating strong inherent robustness to noise in deeper CNN models. However, this observation is derived from a single experimental run without multi-seed evaluation or formal statistical significance testing (e.g., McNemar’s test or DeLong’s test), and the noise augmentation protocol warrants further standardization; consequently, this finding should be regarded as preliminary and interpreted with caution pending replication under controlled experimental conditions.

**Table 5:** Comparison of performance (accuracy and F1-score) between original and noise-added datasets (CNN 4-block).

Pooling method	Accuracy NM (%)	Accuracy NM+noise (%)	F1-score NM (%)	F1-score NM+noise (%)
Max Pooling	94.79	90.69	94.79	90.68
Average Pooling	89.47	88.69	89.44	88.63
Hybrid Pooling	92.90	93.68	92.90	93.68

Notably, the Hybrid pooling method reveals remarkable noise resilience in this deeper architecture scenario, improving its relative standing compared to the 3-block CNN case. Its accuracy and F1-score remain impressively high under noisy conditions, at 93.68%, even surpassing Max pooling’s performance in the presence of noise. This suggests that deeper CNN architectures might enhance Hybrid pooling’s adaptive capabilities, effectively compensating for noise interference. Conversely, average pooling consistently shows the weakest performance across both noise-free and noisy scenarios (accuracy dropping from 89.47% to 88.69%, and F1-score from 89.44% to 88.63%). Thus, Average pooling, while stable, consistently underperforms relative to other methods, particularly in challenging, noisy conditions. Overall, these findings emphasize the effectiveness and robustness of Hybrid pooling within deeper CNN architectures, underscoring its strong potential as a clinically practical pooling method for ultrasound breast cancer imaging.

**Figure 2** provides a comprehensive analysis of the convergence behaviors for CNN architectures employing hybrid pooling under various experimental conditions (CNN depth and dataset noise levels). For the 3-block CNN architecture, training and validation loss steadily decreased, accompanied by an increase in accuracy, indicating stable learning dynamics on both clean (**Figure 2a**) and noise-added datasets (**Figure 2b**). However, the presence of 10% speckle noise slightly delayed convergence and resulted in a marginally lower final validation accuracy, highlighting the model’s sensitivity to noisy conditions. Notably, the deeper 4-block CNN model (**Figure 2c, d**) exhibited faster convergence, more pronounced accuracy improvements, and lower final loss compared to the 3-block model. Nevertheless, it showed signs of potential overfitting, particularly evident in the divergence between training and validation loss curves toward later epochs, especially in the presence of noise. These findings suggest that deeper CNN architectures enhance classification accuracy and convergence speed significantly but require careful regularization to maintain generalization performance under noisy clinical conditions. Quantification of this overfitting gap by reporting training versus validation accuracy and loss at the best-performing epoch alongside systematic evaluation of mitigation strategies such as weight decay, data augmentation, and early stopping, is acknowledged as a limitation of this preliminary study and is designated as a concrete direction for future work.

The results obtained in this preliminary study highlight the potential of an adaptive entropy-weighted hybrid pooling method in improving the diagnostic performance of CNN-based breast ultrasound classification. Specifically, the hybrid pooling strategy consistently outperformed average pooling and was competitive with max pooling, particularly demonstrating superior feature extraction capabilities in shallow CNN architectures. Importantly, the deeper CNN (4-block) architectures significantly enhanced overall performance metrics, noise robustness, and convergence speed, confirming the advantages of increased model depth. However, deeper models also exhibited signs of overfitting, suggesting that additional regularization or fine-tuning strategies may be necessary to optimize generalization performance further. These findings emphasize that the proposed entropy-driven adaptive hybrid pooling approach effectively balances detailed feature preservation and noise reduction, making it particularly promising for practical clinical applications, although further validation with larger and more diverse datasets remains necessary. To contextualize the observed overfitting particularly visible in the 4-block CNN loss curves (**Figures 2c, d**) it is important to note that several strategies were deliberately withheld in the preliminary 12-epoch comparison (**Figure 2**) to ensure a clean, unconfounded comparison of pooling mechanisms; these were subsequently introduced in the final multi-seed protocol (Section 2.2.1). In the preliminary comparison: (i) Data augmentation (random flipping, rotation, scaling) was not applied, as it would have introduced variability orthogonal to the pooling comparison; (ii) early stopping was not used, and all models were trained for the full 12 epochs with convergence confirmed visually by stabilization of loss and accuracy curves by epoch 10; (iii) Cross-validation was not performed, as the fixed stratified 80/10/10 split was sufficient for comparative benchmarking in this preliminary context. Future work will systematically evaluate weight decay (L2 regularization) and k-fold cross-validation to quantify generalization gaps more rigorously and mitigate overfitting in deeper architectures, building on the early-stopping protocol already adopted for the multi-seed experiments (Section 2.2.1).

### 3.3 Complete noise-robustness

**Table 3** presents the unified noise-robustness grid covering all four train/test protocol combinations across two resolutions and two pooling strategies (Max and Hybrid), reported as mean ± SD over three random seeds (14 total experimental conditions).

At 64×64 resolution, Hybrid pooling achieves the highest clean-condition accuracy (0.9398 ± 0.0172), and critically, it also delivers the best performance under the most challenging cross-condition scenario (Mixed=>Noisy10%: 0.9106 ± 0.0032), outperforming Max pooling in the same condition (0.8858 ± 0.0280). This demonstrates that mixed-training augmentation effectively bridges the generalization gap: comparing clean-only training versus mixed training under noisy test conditions (Clean=>Noisy10% vs. Mixed=>Noisy10%), Hybrid pooling improves from 0.8950 ± 0.0145 to 0.9106 ± 0.0032, while Max pooling similarly improves from 0.8780 ± 0.0178 to 0.8858 ± 0.0280.

At 128×128 resolution, the Clean=>Clean condition was not evaluated (indicated by “-”), as higher-resolution training without noise augmentation was computationally reserved. Nevertheless, Hybrid pooling consistently outperforms Max pooling across all three evaluated conditions, with the largest margin observed in Mixed=>Clean (0.8721 ± 0.0052 vs. 0.8525 ± 0.0091). Notably, the standard deviations for 128×128 are generally comparable to or smaller than those at 64×64, indicating stable training across seeds.

Overall, the results confirm that Hybrid pooling with mixed-ratio training (60% noisy: 40% clean, following Jiang et al. (17)) provides the most robust and consistent performance across all evaluated conditions and resolutions.

### 3.4 Resolution ablation

Counter-intuitively, increasing input resolution from 64×64 to 128×128 degraded accuracy across all noise protocols and both pooling strategies (e.g., Hybrid clean=>noisy dropped from 89.50 ± 1.45% at 64×64 to 83.96 ± 1.91% at 128×128, Max dropped from 87.80 ± 1.78% to 76.90 ± 3.37%). We attribute this to insufficient architectural depth relative to the enlarged spatial feature maps: the 3-block design was calibrated for 64×64 inputs, and at 128×128 the global-average-pooled vector aggregates over a larger spatial domain without a compensatory increase in receptive field or channel capacity. Notably, Hybrid pooling consistently preserved a larger margin over Max at 128×128 (Δ ≈ 7% at clean=>noisy), suggesting that its entropy-adaptive weighting partially compensates for the capacity mismatch. Deeper 4- or 5-block variants at 128×128 are designated as future work.

### 3.5 Interpretability analysis

Grad-CAM and Score-CAM heatmaps were generated for all 902 test images for each experiment. Qualitatively, Hybrid-pooling activations concentrate more tightly on lesion boundaries and internal heterogeneous textures, whereas Max-pooling activations exhibit more diffuse, occasionally off-lesion peaks. α(R) entropy maps (**Figure 4**) confirm that the adaptive weight approaches 1 (max-like behavior) near lesion margins and falls toward 0 (average-like behavior) over homogeneous parenchyma, providing a mechanistic visualization of the entropy-weighted strategy.

### 3.6 Computational efficiency

Inference time for the 3-block models was measured on Google Colab Pro+ using time.perf_counter() with batch size = 1, averaged across the 902-image test set for each of three seeds. Per-image inference for Hybrid pooling was 2.18 ± 0.09 ms (64×64) and 3.54 ± 0.20 ms (128×128); Max pooling was 2.18 ± 0.14 ms and 2.28 ± 0.09 ms, respectively. Total parameter counts were 61,392 (Hybrid) and 61,362 (Max), an overhead of only 30 learnable parameters across the three pooling layers (10 per layer), which adjust the entropy scaling (Step 1), the α gating (Step 2), and the max/average mixing weights (Step 3). These latencies are well within clinical AI-assisted workflow tolerances (<500 ms per image). The source code and model weights are publicly available at https://github.com/gitonjun/ESUTNet-HybridPooling.

### 3.7 Limitations

Several limitations of this study should be acknowledged. (i) Multi-seed evaluation (three seeds: 42, 123, 456) was applied only to the 3-block CNN experiments (**Tables 2** and **3**); the 4-block CNN results (**Tables 4** and **5**) were obtained from a single training run with no fixed random seed, so no variability estimates are available for that configuration. Statistical power therefore remains limited, and replication with ≥5 seeds across all architectures, together with multiple independent data splits, is a priority for follow-up work. (ii) The training/validation/test partition is image-wise rather than patient-wise because the Kaggle source repository does not provide patient-level identifiers, which may yield optimistic estimates if images from the same patient span multiple splits. (iii) Evaluation is restricted to a single publicly available breast ultrasound dataset (16); external validation on multi-center ultrasound cohorts with heterogeneous scanner, transducer, and population characteristics is required. (iv) Input resolution is capped at 128×128 within a fixed 3-block architecture, and the observed performance degradation at 128×128 suggests the need for deeper variants. (v) Only 10% speckle-noise intensity was tested; a wider noise-level sweep and physics-based simulations (Field II, k-Wave) are left to future work. (vi) Pairwise AUC significance testing relied on the DeLong non-parametric approach (24) and ROC confidence bands were computed from 3 seeds (mean ROC ± 1 SD); larger seed counts would provide tighter bands.

Clinical integration. The proposed entropy-weighted hybrid pooling layer is a drop-in replacement for conventional pooling operations, requiring no changes to upstream CNN blocks or downstream classifiers. This facilitates integration into existing PACS-based computer-aided diagnosis (CAD) pipelines, where the model can operate on DICOM-formatted ultrasound frames as a radiologist second-opinion tool. In a typical deployment, acquired B-mode images are routed from the ultrasound workstation to a PACS node, resized and normalized by a preprocessing service, and classified by the VMC-Net backbone; the returned probability and Grad-CAM overlay are displayed alongside the original image in the radiologist’s viewer. Because per-image inference is below 5 ms on a single GPU, the model is compatible with near-real-time workflows and batch overnight processing. Potential clinical roles include (a) prioritization of suspicious cases for expedited review, (b) a second-reader sanity check to reduce false negatives in high-volume screening, and (c) structured reporting assistance by supplying provisional BI-RADS-like categories. Prospective, reader-study-based validation with institutional PACS integration and PDPA/HIPAA-compliant data handling is a necessary next step before any clinical deployment.

## 4 Conclusion

This preliminary exploration rigorously investigated the effectiveness of an entropy-weighted hybrid pooling method in CNN architectures for ultrasound-based breast cancer detection. The adaptive pooling approach dynamically integrates the advantages of Max and Average pooling, leveraging local image complexity as measured by Shannon entropy to optimize the balance between noise suppression and detailed feature retention. Experimental outcomes indicated that the proposed hybrid pooling method consistently achieved superior performance compared to conventional Average pooling and offered highly competitive though not universally superior results relative to Max pooling, with its clearest advantage demonstrated within shallow CNN architectures and noisy training conditions. Furthermore, deeper CNN configurations substantially improved overall accuracy, robustness to noise, and convergence speed, attaining AUC scores and classification metrics notably higher than their shallow counterparts. Nonetheless, these deeper models exhibited signs of overfitting, emphasizing the necessity of additional regularization or optimization strategies for maintaining strong generalization in clinical settings. From a clinical deployment perspective, the proposed pooling mechanism functions as a drop-in replacement within standard CNN architectures, enabling integration into existing computer-aided diagnosis workflows without requiring full model retraining, thereby lowering the barrier to clinical adoption. Collectively, these promising findings underscore the proposed entropy-driven pooling method’s potential as a robust, adaptable, and clinically relevant approach for improving ultrasound-based breast cancer diagnostics, warranting further validation through larger, multicentric datasets, interpretability analyses, and collaborative clinical evaluations. Future work should also include visualization of the entropy-derived α(R) weighting maps overlaid on ultrasound images alongside Grad-CAM saliency maps, to verify whether hybrid pooling directs attention toward clinically relevant regions such as lesion boundaries relative to max pooling. Unlike the prior preliminary report, which relied entirely on single-run evaluation, the 3-block noise-robustness grid in this revision (14 conditions; **Table 3**) was repeated over three random seeds with early stopping, ReduceLROnPlateau scheduling, paired statistical comparisons, and DeLong tests on AUC. The 4-block experiments (**Tables 4** and **5**) remain single-run and are designated for multi-seed replication in future work. Under this protocol, hybrid pooling consistently outperformed max pooling across the majority of the multi-seed 3-block conditions, with its clearest advantage emerging under noise-robustness protocols and higher-resolution inputs. Future work will extend seed counts to ≥5, integrate transfer learning from ImageNet-pretrained backbones, test deeper 4- and 5-block variants at ≥224×224, and conduct external validation on multi-center datasets with patient-level splits.

## Data Availability

The dataset is publicly available on Kaggle (16). The source code and model weights are available at https://github.com/gitonjun/ESUTNet-HybridPooling.
